# Factors Associated With Oral Cancer Adverse Outcome at the Rwanda Military Hospital, a Retrospective Cross-Sectional Study

**DOI:** 10.3389/froh.2022.844254

**Published:** 2022-03-18

**Authors:** Halifa Ndayisabye, Albert Ndagijimana, Emmanuel Biracyaza, Aline Umubyeyi

**Affiliations:** ^1^Epidemiology and Biostatistics Department, School of Public Health, University of Rwanda, Kigali, Rwanda; ^2^Department of Clinical and Public Health Services, Ministry of Health, Government of Rwanda, Kigali, Rwanda; ^3^Programme of Sociotherapy, Prison Fellowship Rwanda, Kigali, Rwanda

**Keywords:** oral cancer, management, behavioral factor, oral cancer treatment, adverse outcome

## Abstract

Oral cancer (OC) is one of the most common cancers that remain global public health concerns in low- and middle-income countries. The epidemiology of OC in Africa endures uncertain. Earlier reports suggested a relatively low incidence of OC among Africans. Acting on behavioral factors and setting early diagnosis and treatments of OC can tremendously reduce morbidity and mortality related to it. This study determined factors associated with the OC adverse outcome and death in the Rwanda Military Hospital. A cross-sectional study was conducted among 311 medical records of patients who consulted in the Oral and Maxilla Facial Department between January 1, 2007 and December 31, 2019. Associated factors were estimated by use of odds ratios (OR) with their 95% confidence intervals (CI) in bivariate and multivariate logistic regression analyses to estimate predictors of an OC adverse outcome and death. Almost three quarters of the participants were from rural areas (*n* = 229, 73.6%) and alcohol users (*n* = 247, 79.1%). Concerning primary site infection, 54.02% of the participants had the intra-oral cavity within the past 5 years. Muslims had greater odds to experience an OC adverse outcome and death [aOR = 6.7; 95% CI (3.8–11.9), *p* < 0.001] than the Catholics. Those with no formal education significantly had greater likelihoods to have an OC adverse outcome and death [aOR = 2.6; 95% CI (1.3–5.3), *p* = 0.005] than those with higher education or university. Those with primary education had greater likelihoods [aOR = 1.8; 95% CI (1.1–3.1), *p* = 0.03] to have an OC adverse outcome than those with higher or university education. Those with oral hygiene had less risk to have an OC adverse outcome and death [aOR = 0.2; 95% CI (0.0–0.9), *p* = 0.039] than their counterparts. Using multi-sectorial approaches, including policy makers, clinicians, and researchers from public and private institutions, may be of an added value to promote clinical research on OC for earning knowledge, contributing to combat risk behaviors and improve the population's information and education on OC prevention.

## Background

The epidemiology of oral cancer (OC) indicated that this disease is the 11th common disease worldwide, and two thirds of the cases are from low- and middle-income countries (LMICs) [1]. Also, the OC in the African setting is poorly documented. Earlier reports suggested a relatively low incidence of OC among Africans [2]. So, the OC refers to a group of diseases characterized by the uncontainable growth of cells that attack and destroy the neighboring tissues. Furthermore, there is an indication that the incidence of OC is rising in LMICs in direct proportion to the increase of risky behaviors, mainly tobacco and alcohol use; this results in high prevalence of OC [1, 3]. The OC is also defined as a pathogenous neoplasia, which appears on the lip or oral cavity. This disease includes cancer of the lips, cheeks, tongue, floor of the mouth, hard and soft palates, pharynx, and sinuses that can be life-threatening if not diagnosed and treated early [4, 5]. The most common type of OC is the cancer of the oropharynx or throat that often affects the tongue, mouth, and tonsils [6]. These cancers hamper the quality of life, while patients with cancer breathe, eat, and speak. Since each part of the oral cavity is unique, this cancer includes a wider range of cancers that are complex and managed differently [7]. Worldwide, every year, more than 500,000 people have OC, and more than a half of them die of these cancers within the first 5 years of diagnosis [8, 9]. These diseases are also highly prevalent in men who are mostly exposed to the risk behaviors like smoking and alcohol use [10, 11].

Every patient suffering from OC presents different symptoms and levels of pain. Staging and diagnosing the disease are important for tailored and adequate treatment for each patient's specific OC type [12]. The types of OC based on stages can be squamous cell carcinoma (SCC); above 90% of all cancers occur in oral parts as squamous cell carcinomas [13]. Verrucous carcinoma—~5% of all tumors that orally occur as tumors called verrucous carcinoma—this type grows slowly and is also built in squamous cells [6]. There is also cancer of salivary gland that comprises many types of carcinoma resulting in lymphoma [12]. Oral cancers can also start from the lymph cells and is referred to as lymphomas [13]. Benign tumors of the oral cavity can be of several types, such as non-cancerous tumors and tumor-like conditions that may develop in the oral cavity and sometimes in oropharynx [14]. Contributing predictors of the process of seeking medical attention include unawareness of signs and symptoms, disavowal, and lack of knowledge about OC [15].

Moreover, factors like smoking, untreated oral disease, alcohol, and HPV contribute more to the burden of OC in different regions of the world [9, 15], but, in the Eastern region of Africa, Uganda was ranked high in global alcohol consumption, and it is catching up on tobacco product use. So, this indicates that there is a high incidence of OC cases in the Eastern African countries [16]. Additionally, previous studies reported that two thirds of patients with OC are diagnosed at an advanced stage of OC, and this indicates that the delay may lead to a high mortality and morbidity [17]. Although the OC has been highly prevalent in Asia, this disease has also been a public health burden in the Eastern Africa, such as in Kenya, Uganda, and Tanzania, where its prevalence varies from 2 to 3.6% [16, 18, 19]. Smoking alone has claimed an estimated 71% of lives that died and were diagnosed with OC in developed countries against about 37% in LMICs [11]. In the same light, in LMICs, alcohol consumption accounts for 33% of new cases and 14% of deaths [9, 10]. Early detection and treatment of OC remain important health policy, and this improves survival and cost-effectiveness for health services, and lessens disfigurement [15, 20]. OC treatment options include surgical excision, radiotherapy, and chemotherapy, using one or a combination of all, but all these therapies may be provided in combination, depending on the stage of the cancer [4]. A greater proportion of patients usually seek for care when the disease is already in its late stages, which decrease their probability of being cured at 30% [21–23]. Also, detection of OC at the early stage is effectively important to enhance psychosocial outcomes and quality of life [20].

In Sub-Saharan African countries, high prevalence of mortality due to poor knowledge of OC management and other health issues has been debatable [24]. This increase is due to the harmful effects of changes in lifestyle and the increase of the emergent new diseases that are often associated with behavioral influences [2, 24, 25]. Then, the Government of Rwanda has made efforts to combat OC through various interventions in terms of treatment and prevention of cancers. These programs have positively resulted in health promotion; however, OC remains a national public health burden. The WHO report of 2017 states that mortality due to OC is up surging, and Rwanda has recently been ranked at the 28th position worldwide among countries with a high incidence of OC [26]. As a result, there is a need to adequately document more literature on OC to guide decision-making and proper management of OC at different levels. Our results will provide concrete evidence to suitably understand the associated factors of OC so as to inform policy makers or decision makers. Therefore, this study investigated the factors associated with the OC adverse outcome and death in the Rwanda Military Hospital (RMH).

## Methods

### Study Design

A retrospective hospital-based cross-sectional study was designed to estimate the proportion of oral cancer case fatality at Rwanda Military Hospital (RMH) and determine the factors associated with the oral cancer adverse outcome and death among patients seeking healthcare services at the RMH.

### Study Setting

This study was conducted at the Rwanda Military Hospital (RMH), which is a public health facility situated in Kigali City, Rwanda. This health facility is the only military hospital and one of the five national referral hospitals in Rwanda [27, 28]. The hospital has the bed capacity of two thousand patients, and it contains more than twenty departments with an Oral and Maxilla Facial Surgery department where cases of OC are treated. The patient data from this department are recorded on registers and electronic medical records. The hospital is the sole one providing such oral cancer services in Kigali City and renders assistance to patients from the whole country.

### Study Population and Sampling

This study targeted patients presenting diagnosis of oral cancer at RMH between January 1, 2007 and December 31, 2019. The study included the participants who represented signs and symptoms of oral cancer and visited the Oral Maxilla Facial Surgery department of this hospital. The patients who were diagnosed with OC were provided with healthcare interventions or treatments. The current study was only conducted among the patients who were at the fourth stage and who were under the treatments such as surgery and radiation. The medical records of all the patients with other diagnosis other than OC at the fourth stage were excluded from this study. This department recorded a total of 311 patients with OC as the principal diagnosis at the fourth stage; hence, all the diagnosed patients with OC were exhaustively included in this study.

### Procedures

Data were collected by trained health providers for 2 weeks in January 2020. The participants from the study had their health records at the abovementioned department. All the data were extracted from the patient registries from this department. Records of patients with cancer were reviewed to extract clinical information and a demographic profile that were routinely recorded in the department that provide care for patients with OC. Among the patients that were contacted to complete missing information, no patient refused to take a part in in the study. However, for the missing information, the researchers contacted the patients or their guardians to provide more details. These patients were contacted over the phone to collect the missing information about their health status. Missing data and follow-up data were retrieved and updated from the case records. Besides, all discrepancies were appropriately checked and corrected by referring back to the original patients' records. About 5% of data validation was applied by second data entry personnel to assess accuracy of the data. All rates of missing information were reported for each question of the questionnaire. A paper-based checklist was used for data collection. Furthermore, the researcher inputted the data into Microsoft Excel 2010 and then later exported them to STATA version 13.0 for statistical analysis.

### Study Variables

The main outcome variable was “an oral cancer adverse outcome and death” in which we refer to as a patient experiencing an adverse outcome and death or not. This binary variable was coded as “1 = Yes” and “0 = No.”

Independent variables were investigated. Those factors included sociodemographic variables and behavioral characteristics of the patients. Among the sociodemographic variables, type of residence was coded as follows (0 = rural, 1 = urban), gender of the patient grouped as (1 = male, = female), education categorized as (1 = higher/university, 2 = secondary education, 3 = primary, 4 = no formal education), while ages of the patients were grouped into (1 = 0–20 years, 2 = 21–40 years, 3 = 41–60 years, 4 = 61–80 years, 5 = 81 years and above). Furthermore, marital status was coded as (1 = married, 2 = living with a partner, 3 = widowed, 4 = divorced, 5 = no longer living together or separated) and religion as (1 = Catholics, 2 = Muslim, 3 = others). Concerning behavior-related factors, the alcohol use was categorized as (0 = no, 1 = yes), smoking or tobacco use as (0 = no, 1 = yes), and oral hygiene as (0 = no, 1 = yes).

### Data Analysis

All descriptive and analytical statistical analyses were performed using STATA software version 13. Bivariate logistic analyses were performed to indicate the associations between dependent and independent variables. The significant variables in bivariate analyses were exported into multivariate logistic regression models for determining associated factors of an OC adverse outcome and death. The odds ratios were used to assess associated factors. The 95% confidence intervals and statistical significance levels of *p* < 0.05 were considered.

### Ethics

The Helsinki declaration regarding the ethical principles for medical research that involve human participants was taken into consideration as recommended [29, 30]. Therefore, the study was reviewed and approved by the Institutional Review Board of the Rwanda Military Hospital with the reference number (Ref.: RMH IRB/034/2019). The participants were contacted to provide the permission to use the information from their records. Privacy and confidentiality were ensured by not providing the identifiable information from the patients. All data were anonymously kept.

## Results

### Sociodemographic Characteristics of Study Participants (*N* = 311)

Our findings show that the average age was 50.6 years (SD = 20.2). Out of 311 records, the majority (*n* = 111, 35.7%) were aged 41–60 years, were males (*n* = 171, 56%), and were married (*n* = 256, 82.3%). Near two thirds of the study participants (*n* = 212, 68.2%) were Catholics, and about a half had at least primary school education (*n* = 147, 47.3%). Overall, the participants came mostly from Eastern Province (*n* = 116, 37.3%) of Rwanda, with a greater proportion of them residing in rural areas (*n* = 229, 73.6%). The results also demonstrated that a good proportion of the participants came from middle-income class (*n* = 259, 83.3%) who were likely to brush their teeth at least one time daily (*n* = 227, 73%). Additionally, more than 50% of them never smoked tobacco (*n* = 183, 58.8%) even though most of them had ever consumed a certain quantity of alcohol (*n* = 246, 79.1%) (Table 1).

**Table 1:** Sociodemographic characteristics of participants.

**Variables**	**Frequency (*N* = 311)**	**Percentage**
**Age**		
0–20 years	26	8.4
21–40 years	67	21.5
41–60 years	111	35.7
61 years and above	107	34.4
**Sex of patient**		
Female	140	44
Male	171	56
**Marital status**		
Never been married	55	17.7
Ever been married	256	82.3
**Religion**		
Catholic	212	68.2
Muslim	10	3.2
Others (e.g., Adventists, Witnesses of Jehovah, Anglicans, ADEPR)	89	28.6
**Educational level**		
Higher education/ University	12	3.9
Secondary education	62	19.9
Primary	147	47.3
No formal education	90	29
**Place of residence**		
Urban	82	26.4
Rural	229	73.63
**Wealth index**		
High	5	1.6
Middle	259	83.3
Poor	47	15.1
**Oral hygiene**		
No	84	27
Yes	227	73
**Smoking**		
No	183	58.8
Yes	128	41.2
**Alcohol use**		
No	65	21
Yes	246	79.1

*N, Frequency, % had been shown to indicate the level of occurrence; ADEPR, Association des Eglises de Pentecoteau Rwanda*.

### Clinical Characteristics of the Study Participants

The clinical results indicated that the majority of the study participants (*n* = 166, 53.4%) were diagnosed with intra oral cavity within the past 5 years. More patients (*n* = 238, 76.5%) accepted OC treatments at our RMH than the few (*n* = 73, 23.4%) who refused (Table 2).

**Table 2:** Clinical characteristics of the study participants.

**Variables**	**Frequency (*N* = 311)**	**Percentage**
**Primary site infections**		
Intra oral cavity	166	53.4
Extra oral cavity	98	31.5
Mandible	36	11.6
Maxillary, tonsil, and tongue	11	3.5
**Accept initiation to OC treatments**		
Yes	238	76.5
No	73	23.5

### Treatment Status for Patients With Oral Cancer

**Figure 1:**
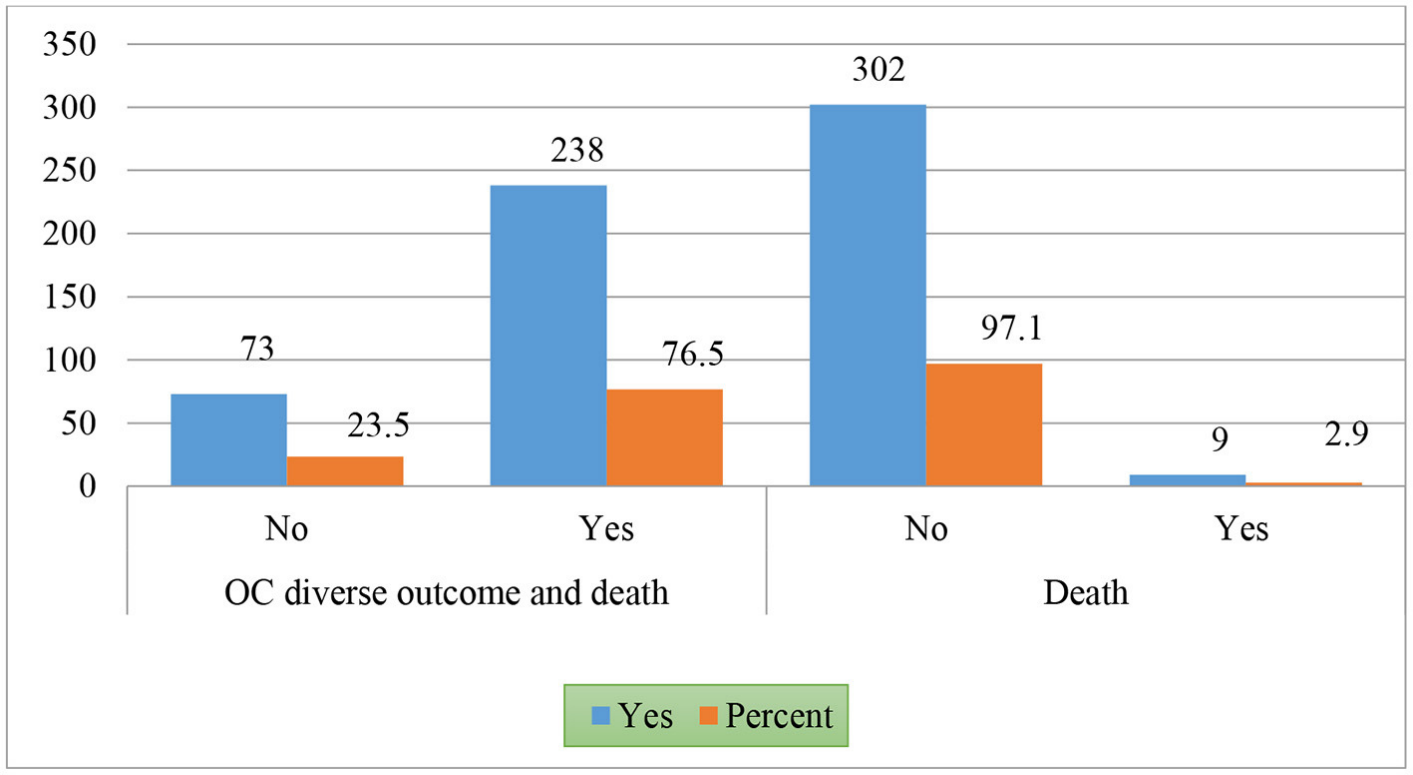
An oral cancer adverse outcome and death.

Among 311 patients, the results indicated that 9 patients (2.9%) died, but the majority survived (*n* = 302, 97.1%) (Figure 1).

### Association Between the Oral Cancer Adverse Outcome and Death and Sociodemographic and Behavioral Factors

Factors associated with this outcome included educational background, province, and religion. Additionally, these factors were significantly associated with an OC adverse outcome and death. However, the factors, such as age, gender, marital status, residence, primary site of infection, and wealth index, were not found to be significant. Our results from bivariate analysis showed that the patients with no formal education have almost three times the risk of having the OC adverse outcome and death compared to the patients with higher education [OR = 2.3, 95% CI (1.2–5.2), *p* = 0.011], while those with secondary education were nearly three times more likely to experience an oral cancer adverse outcome and death [OR = 2.7, 95% CI (2–3.7), *p* < 0.001]. Furthermore, the participants with primary education had nearly 2 times more exposed to an OC adverse outcome and death [OR = 1.6, 95% CI (1.1–2.2), *p* = 0.002] than those with higher education. Furthermore, the results indicated being Muslims was significantly associated with an OC adverse outcome and death. These results showed that Muslims had almost 9 times the risk to experience the oral cancer adverse outcome and death [OR = 8.6; 95% CI (1.5–49.4), *p* = 0.016] than the Catholics (Table 3).

**Table 3:** Association between the oral cancer adverse outcome and death and sociodemographic and behavioral factors.

**Values**	***N* (%)**	**OR**	**95% CI**	***P*-value**
**Age**				
0–20 years	21 (8.8)	1		
21–40 years	55 (23.1)	0.8	[0.1–4.5]	0.776
41–60 years	79 (33.2)	0.1	[0.01–1.3]	0.076
61 years and above	83 (34.9)	0.3	[0.03–1.9]	0.183
**Sex**				
Female	99 (41.6)	1		
Male	139 (58.4)	1.1	[0.3–3.9]	0.972
**Marital status**				
Never been married	69 (29)	1		
Ever been married	169 (71)	0.4	[0.1–1.7]	0.225
**Religion**				
Catholic	160 (67.2)	1		0.023^*^
Muslims	8 (3.4)	2.8	[1.4–5.7]	0.005^*^
Others (e.g., Adventists, Witnesses of Jehovah, Anglicans, ADEPR)	70 (29.4)	0.5	[0.2–1.1]	0.061
**Education**				
Higher education/University	6 (2.5)	1		<0.001^***^
Secondary education	50 (21)	2.7	[1.9–3.7]	<0.001^***^
Primary education	117 (49.2)	1.6	[1.1–2.2]	0.002*^*^
No formal education	65 (27.3)	2.6	[1.2–5.2]	0.011^**^
**Residence**				
Urban	58 (24.4)	1		
Rural	180 (75.6)	0.4	[0.1–1.7]	0.224
**Oral hygiene**				
No	67 (28.2)	1		
Yes	171 (71.8)	0.5	[0.4–0.9]	0.014^*^
**Smoking**				
No	137 (57.6)	1		
Yes	101 (42.4)	0.2	[0.02–1.4]	0.099
**Alcohol use**				
No	51 (21.4)	1		
Yes	187 (78.6)	0.9	[0.2–4.6]	0.921
**Primary site of infection**				
Intra oral cavity	133 (55.9)	1		
Extra oral cavity	68 (28.6)	1.8	[0.4–7.3]	0.421
Mandible	29 (12.2)	1.2	[0.2–10.8]	0.889
Maxillary, tonsil and tongue	8 (3.4)	1.6	[0.6–4]	0.321

* *Statistically significant with p < 0.05*.

** *Statistically significant with p < 0.01*.

*** *Statistically significant with p < 0.001*.

### Multivariate Association Between the Oral Cancer Adverse Outcome and Death and Sociodemographic and Behavioral Factors

Findings of this study showed that the religion, level of education, and hygiene were significantly contributors of the OC adverse outcome and death. For instance, Muslims were almost 7 times more likely to have an OC adverse outcome and death [aOR = 6.7; 95% CI (3.8–11.9), *p* < 0.001] when compared to the Catholics. Furthermore, those who had no formal education were almost 3 times more likely to have an oral cancer adverse outcome and death [aOR = 2.6; 95% CI (1.3–5.3), *p* = 0.005] when compared to those with higher education. Results indicated that those who attended secondary schools had almost 2 times greater risk to develop an OC adverse outcome and death [aOR = 1.8, 95% CI (1.1–3.1), *p* = 0.003] when compared to those who had higher education or university. The results also showed that those with oral hygiene were less likely to experience the OC adverse outcome and death [OR = 0.2, 95% CI (0.03–0.9), *p* = 0.039] (Table 4).

**Table 4:** Multivariate analysis for factors associated with oral cancer treatment.

**Values**	**aOR**	**95% CI**	***P*-value**
**Religion**			
Catholic	1		<0.001^**^
Muslims	6.7	[3.8–11.9]	**<**0.001^**^
Others (e.g., Adventists, Witnesses of Jehovah, Anglicans, ADEPR)	1.2	[0.7–1.9]	0.52
**Oral hygiene**			
No	1		
Yes	0.2	[0.03–0.9]	0.039^*^
**Level of education**			
Higher education/University	1		0.004^*^
Secondary education	1.8	[0.9–3.4]	0.093
Primary education	1.8	[1.1–3.1]	0.03^*^
No formal education	2.6	[1.1–5.3]	0.005^*^

* *Statistically significant at p < 0.05*.

** *A statistical significance level at p < 0.001*.

## Discussion

The purpose of this epidemiological study was to determine the factors associated with the OC adverse outcome and death in the Rwanda Military Hospital (RMH). Although the case fatality was not documented in the other parts of Africa, ~2.9% of the patients with OC who were consulted at the RMH died. This case fatality rate is higher than that reported from Uruguay [31]. Our results also revealed that the mortality rate of OC is lower than the mortality in the African countries (such as Uganda, South Africa, Egypt, Nigeria, Kenya, Tanzania) and Asian countries [24, 32–34].

Among these patients, the compromised patients with four-stage OC, the study deducted that factors, such as education, poor oral hygiene, and religion, were significant predictors of the OC adverse outcome and death. Our results are similar to the findings from prior studies [11, 35]. Essentially, this study found that the educational level has a key part to play in the OC adverse outcome and death for the patients diagnosed this health burden associated with the changes of daily living. Those with no formal education, secondary education, and primary education presented greater odds to experience the OC adverse outcome and death when compared to those with higher education or university education. These findings collaborated with previous studies that conveyed that education plays a great role in preventing and treating patients from OC [4, 36, 37]. This could be because educated people are well-informed on importance of seeking healthcare services at the early stage of cancer when compared to those with no informal education are less likely to have OC adverse outcome and death. Another explanation could be since educated people have a better social status within their communities and a good support system, which facilitates access to treatment as seen in other types of cancers. These results are in accordance with prior studies [5, 38, 39].

Even though the studies in United States identified that the stage of cancer, age, overall health situation, and primary site infected affect the treatment success of oral cancers [23, 40, 41], the results from our study challenged the prior results that revealed no significant association between the OC adverse outcome and death and factors like age of the patient, stage of OC, and the primary site infected. Another important explanation is that only the patients with stage-four OC were studied according to the sampling frame used. Imperatively, there were also confounders like the patients' HIV and Human Papilloma Virus status that were not considered because the data were not collected or the patients were not screened for the ailments.

Furthermore, the study found that religion plays an important part in determining if individuals are treated or otherwise from oral cancer. Our results revealed that the Muslims were more likely to have greater likelihood of having an OC adverse outcome and death when compared to the Catholics, which controverted the preceding studies that documented no sociodemographic characteristics contribute to a low rate of patients with OC who accept OC treatment initiation or delay [42]. Even though it is difficult to explain how religion acts on treatment response, the findings are that being a Muslim facilitates someone to recover or not form oral cancer.

This study had numerous limitations. The inability of the study to detect factors associated with oral cancer in terms of an anatomical oral cancer site was probably related to retrospective nature of the study and the fact that it relied on secondary information from a number of variables and observations. Besides, the study was limited to the sample that was available but not big enough to allow generalized findings. This suggests further studies with more variables and observations to investigate factors associated with unspecified oral cancer sites among cases recorded at RMH. For the latter; the topic for this study shows that the related data have the most of challenges regarding completeness and consistency in data collection. This handicapped this study to clearly and objectively estimate the prevalence of the oral cancer adverse outcome and death. The registry did not provide complete information on certain vital factors such as HPV and HIV status. This confounded the study and might have affected the true estimate of the oral cancer adverse outcome and death.

## Conclusion

To conclude, the mortality in this study among the patients diagnosed with OC in the fourth stage of the OC represented an elevated OC adverse outcome and health that seem to be associated with the major factors, such as education, religion, and oral hygiene. Hence, these parameters need to be taken into account for the individualized therapy management of patients with OC, and more efforts are important to bring light on this burning sector of public health. Based on the nature of this study, we recommend to include in the cancer registry an exhaustive list of necessary variables to be able to deeply understand the epidemiology of OC. Additionally, as several previous studies were hospital-based studies and population based-studies are rare, we recommend further epidemiological studies to conduct population-based studies on assessing the burdens of the OC in sub-Saharan countries where this disease remains the public health burden.

## Data Availability Statement

The original contributions presented in the study are included in the article/supplementary material, further inquiries can be directed to the corresponding author.

## Ethics Statement

Ethical approval was provided by the Institutional Review Board, Rwanda Military Hospital in the Republic of Rwanda. After obtaining the authorization to conduct the study from the Ministry of Health through the general director of RMH, the permission to use the medical records was requested from the patients or their guardians, using the telephone registered at the hospital. Confidentiality was ensured.

## Author Contributions

HN had substantial contribution to conception, data collection, and statistical analysis. AU supervised the study, contributed to the study conceptualization, and assisted in data analysis. AN assisted in conceptualization and implementation of the data analysis and interpretation of the results and provided the manuscript feedback. AU and AN played the editorial role of the manuscript. EB drafted the manuscript and contributed to the interpretation of the results and data analysis. All the authors read and approved the final manuscript.

## Conflict of Interest

The authors declare that the research was conducted in the absence of any commercial or financial relationships that could be construed as a potential conflict of interest.

## Publisher's Note

All claims expressed in this article are solely those of the authors and do not necessarily represent those of their affiliated organizations, or those of the publisher, the editors and the reviewers. Any product that may be evaluated in this article, or claim that may be made by its manufacturer, is not guaranteed or endorsed by the publisher.
